# Spontaneous Muscle Bleeding During Oral Anticoagulation Therapy: When Should We Suspect an Underlying Tumor?

**DOI:** 10.3390/hematolrep17050044

**Published:** 2025-08-31

**Authors:** Antonella Mameli, Francesco Marongiu, Mauro Podda, Adolfo Pisanu, Doris Barcellona

**Affiliations:** 1 Hemostasis and Thrombosis Unit, University of Cagliari and AOU of Cagliari, 09124 Cagliari, Italy; francesco.marongiu@unica.it (F.M.); doris.barcellona@unica.it (D.B.); 2 Emergency Surgery Unit, Department of Surgical Science, University of Cagliari and AOU of Cagliari, 09124 Cagliari, Italy; mauro.podda@unica.it (M.P.); adolfo.pisanu@unica.it (A.P.)

**Keywords:** intramuscular hematoma, anticoagulation, warfarin, soft tissue sarcoma, malignant fibrous histiocytoma, diagnostic delay, imaging

## Abstract

Spontaneous intramuscular hematomas (SMHs) are rare but potentially serious complications of oral anticoagulation therapy. Although often attributed solely to anticoagulant use, such lesions may mask underlying soft tissue sarcomas or paraneoplastic conditions. We report the case of an 80-year-old man on warfarin who presented with a painful thigh mass initially interpreted as a hematoma but ultimately diagnosed as a malignant fibrous histiocytoma (MFH). In addition, we provide a narrative review of published cases, focusing on clinical presentation, diagnostic challenges, imaging strategies, and outcomes. Key pitfalls leading to delayed diagnosis include attribution bias, inadequate imaging, and premature management decisions. Epidemiological data show that while the incidence of SMHs is estimated at 0.5–1.5% among patients on vitamin K antagonists, clinically significant cases are increasingly reported with direct oral anticoagulants (DOACs). Suggested measures include clinical algorithms to prompt imaging and biopsy in persistent masses, validation of magnetic resonance imaging (MRI) criteria, and the establishment of prospective registries, aimed at facilitating earlier recognition of malignant lesions and improving patient outcomes. These strategies may improve early detection of malignancy and optimize care in anticoagulated patients presenting with soft tissue lesions.

## 1. Introduction

Anticoagulant therapy is a cornerstone in the prevention and treatment of cardiovascular and thromboembolic diseases. However, bleeding remains its most feared complication. In recent years, increasing attention has been paid to the temporal association between bleeding events during anticoagulation and subsequent cancer diagnosis [1]. This relationship is often not causal but reflects the ability of anticoagulants to unmask preexisting tumors by inducing bleeding complications [2]. Spontaneous intramuscular hematomas (SMHs) are relatively rare, with a reported incidence of 0.5–1.5% in patients on oral anticoagulation therapy (OAT), particularly with vitamin K antagonists (VKAs) [2]. In the era of direct oral anticoagulants (DOACs), the epidemiology of SMHs appears to be shifting. While several real-world studies suggest a slightly lower incidence compared to VKAs, elderly patients, those with comorbidities such as renal insufficiency or frailty, and patients receiving concomitant antiplatelet therapy remain at high risk. Large registry data indicates that SMHs contribute substantially to hospitalizations and mortality, with retroperitoneal and iliopsoas sites associated with particularly poor outcomes [3]. Beyond their clinical burden, SMHs have diagnostic implications that extend beyond hemorrhagic risk. Evidence suggests that in a subset of patients, muscle bleeding is not merely an adverse drug reaction but a sentinel manifestation of an occult malignancy. The so-called “unmasking phenomenon” has been increasingly reported, in which anticoagulation exposes the fragile vasculature of a hidden tumor, leading to bleeding as the first symptom. This dual interpretation, complication versus sentinel sign, poses major challenges for clinicians and underscores the need for heightened diagnostic vigilance and structured pathways. The unpredictability of such presentations carries major clinical consequences: delayed tumor diagnosis, advanced disease at detection, and worsened prognosis. Given the aging and anticoagulated populations, improving diagnostic vigilance is essential. This clinical report and review present a case of soft tissue sarcoma initially misdiagnosed as a hematoma in a patient treated with warfarin. It illustrates the practical challenges of such cases. In addition, we synthesize the literature on soft tissue masses associated with underlying malignancy, critically assess current diagnostic and management practices, identify shortcomings, and provide recommendations to guide future research.

### 1.1. Case Report

**Figure 1:**
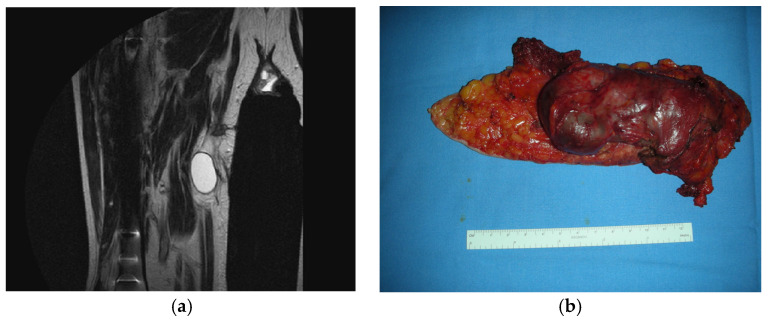
MRI finding (**a**) and gross specimen of malignant fibrous histiocytoma excised in bloc during surgery (**b**).

An illustrative case underscores the diagnostic complexity of spontaneous intramuscular hematomas in anticoagulated patients. An 80-year-old man with permanent atrial fibrillation was on warfarin therapy, since 2018, with stable international normalized ratio (INR) values between 2.0 and 2.5. He presented to the emergency department with sudden pain and swelling of the left thigh, without trauma or recent exertion, beginning 3 weeks before first evaluation. Examination revealed a soft, painful quadriceps mass. Point-of-care ultrasound suggested a large hematoma. Laboratory tests showed INR 2.3 (therapeutic range), normal platelet count, and mildly elevated liver enzyme (AST, ALT, and GGT), with subsequent normalization after withdrawal of concomitant hepatotoxic medication. The mass was initially attributed to spontaneous muscle bleeding, a recognized complication of anticoagulation, and conservative management with rest and compression was initiated. Over the next two weeks, the mass persisted with little size reduction and became increasingly firm. Although clinically stable, the patient developed progressive functional limitation and persistent pain, prompting further imaging. He was admitted for observation and temporary interruption of anticoagulation. Non-contrast CT confirmed an extensive intramuscular hematoma of the vastus lateralis without active bleeding. However, contrast-enhanced MR, performed on 1.5T scanner, with the following sequences, T1-weighted (slice thickness 4 mm), T2-weighted with fat suppression, diffusion-weighted imaging (b = 800 s/mm^2^), and contrast-enhanced T1 with gadolinium (0.1 mmol/kg), revealed a heterogeneous lesion measuring 8.6 × 5.2 × 4.8 cm, with irregular borders, patchy enhancement, internal septations, high T2 signal, restricted diffusion, and an ADC value of 0.72 × 10^−3^ mm^2^/s—features atypical for an organizing hematoma (Figure 1a). Images were independently reviewed by two musculoskeletal radiologists with >10 years’ experience, blinded to the final diagnosis; discrepancies were resolved by consensus (Figure 1a). Based on these findings, a core needle biopsy was performed 10 days after. Histopathology demonstrated a high-grade pleomorphic malignant fibrous histiocytoma (now classified within the undifferentiated pleomorphic sarcoma spectrum). Staging excluded distant metastases. The patient underwent surgical intervention with exeresis of the mass (Figure 1b), followed by adjuvant radiotherapy. Anticoagulation was resumed postoperatively with low-molecular-weight heparin under close monitoring. At six-month follow-up, he remained free of recurrence.

### 1.2. Lessons from the Case

This case illustrates several important principles:

- The necessity of reassessment when the clinical course deviates from expected healing.
- The value of repeat imaging and histological confirmation in uncertain diagnoses.

## 2. The Potential for Anticoagulation to Confound Clinical Judgment

### 2.1. Risk Factors and Clinical Presentation of Spontaneous Muscle Hematomas

Spontaneous muscle bleeding arises from rupture of intramuscular vessels in the absence of trauma, most often in the setting of anticoagulation, platelet dysfunction, or coagulopathies. In anticoagulated patients, hemorrhage is favored by increased vascular fragility due to age or comorbidities (e.g., diabetes, atherosclerosis) [4], excessive anticoagulant activity (e.g., supratherapeutic INR during warfarin therapy) [4], and minor, unrecognized mechanical stresses (e.g., muscle contraction, straining). Risk factors for SMH include advanced age, high-intensity anticoagulation (e.g., INR > 3), concomitant antiplatelet therapy, renal insufficiency, recent surgery or trauma, and active cancer [5]. The most frequently affected sites are the psoas muscle, rectus sheath, and thigh muscles (vastus intermedius, adductor, sartorius) [2,6]. In the oncologic setting, additional mechanisms come into play. Tumor-related vascular fragility reflects the structurally abnormal neo vasculature typical of malignant tissues [1]. Newly formed vessels in sarcomas are often tortuous, dilated, and poorly supported by pericytes, which renders them highly vulnerable to rupture under anticoagulation [2]. Moreover, angiogenic signaling, particularly through VEGF and related pathways, increases vascular permeability and further predisposes to intratumoral bleeding. Another important contributor is the paraneoplastic dysregulation of hemostasis. Many tumors, including soft tissue sarcomas, may induce systemic procoagulant or anticoagulant states by releasing tissue factor, cytokines, or fibrinolytic proteins [3]. While cancer is classically associated with thrombosis, subsets of patients may develop paraneoplastic coagulopathies with acquired deficiencies of clotting factors, platelet dysfunction, or enhanced fibrinolysis. When combined with therapeutic anticoagulation, these abnormalities amplify the bleeding risk and may lead to atypical presentations such as large, spontaneous muscle hematomas. This complex interplay—mechanical vessel rupture in fragile tissue, tumor-driven angiogenesis with unstable vasculature, and systemic paraneoplastic coagulopathy—explains why in some cases muscle bleeding represents not only a complication of therapy but also the first clinical manifestation of an occult malignancy. Recognizing these intertwined mechanisms is essential to move beyond simple attribution to anticoagulant drugs and to appreciate SMH as a possible sentinel sign of cancer. Bilateral or multiple hematomas are uncommon but possible [7]. These lesions usually present localized pain, swelling, and functional impairment and are generally managed conservatively. However, in some cases, they may conceal or mimic neoplastic masses such as soft tissue sarcomas [8].

### 2.2. Managemet Pathways and Multidisciplinary Challenges

A structured management pathway should include three steps: (I) early recognition of red flags during initial clinical evaluation; (II) timely escalation to magnetic resonance imaging (MRI) when the clinical course deviates from spontaneous resolution; and (III) biopsy for persistent or atypical lesions. Such an algorithm minimizes both overtreatment of benign hematomas and delayed recognition of malignant tumors. From a hematology standpoint, anticoagulation management is particularly complex. Temporary discontinuation or bridging with low-molecular-weight heparin (LMWH) may be necessary, but individualized assessment of thrombosis versus bleeding risk is essential. In patients ultimately diagnosed with sarcoma, reintroduction of anticoagulation after tumor control requires close multidisciplinary oversight, as recurrent intratumoral hemorrhage may complicate treatment. When malignancy is suspected, early involvement of multidisciplinary tumor boards, including experts in thrombosis and hemostasis, oncology, radiology, and surgery, can facilitate timely biopsy, staging, and definitive management (surgery ± radiotherapy), while balancing anticoagulation decisions. Patient education is also critical. Informing anticoagulated patients about warning signs such as persistent swelling, pain, or non-resolving bruising may encourage earlier medical evaluation and reduce diagnostic delays.

## 3. Clinical Presentations and Diagnostic Pitfalls

SMHs often present with sudden muscle pain, swelling, and ecchymosis, typically in the iliopsoas, thigh, or rectus sheath [9,10], particularly when the INR is supratherapeutic [9]. Advanced age and comorbidities are associated with poorer outcomes [10].

Case reports and series show that soft tissue sarcomas, especially malignant fibrous histiocytomas (MFH), myxofibrosarcoma, pleomorphic sarcoma, and Ewing sarcoma, may mimic chronic hematomas [8,11,12,13]. Unexpected hemorrhage into a tumor mass and misinterpretation on imaging can delay diagnosis. Studies reported an average delay of 7 months due to misdiagnosis [13,14].

The evaluation of SMHs in anticoagulated patients is fraught with diagnostic pitfalls, often driven by cognitive biases or incomplete workups. A frequent error is attribution bias, the tendency to ascribe soft tissue bleeding solely to anticoagulation, especially when INR values are therapeutic or only mildly elevated. While often correct, this assumption may lead clinicians to overlook other causes, including neoplastic lesions that can mimic or be masked by hematomas [14,15]. A second critical issue is incomplete imaging. In many settings, diagnosis relies on ultrasound or non-contrast computed tomography (CT), both of which may fail to detect underlying malignancy. Ultrasound is commonly first-line given its availability and low cost, but it poorly distinguishes hematomas from solid tumors, especially when deep-seated. Contrast-enhanced CT improves anatomical detail and detects active bleeding yet remains suboptimal for characterizing soft tissue composition. These modalities, although useful in confirming bleeding and defining its extent, lack the sensitivity and specificity needed to identify atypical features suggestive of neoplasia [16]. By contrast, contrast-enhanced magnetic resonance imaging (MRI) provides superior soft tissue characterization and should be used when diagnostic uncertainty or atypical evolution is present [17,18,19]. MRI is considered the gold standard for differentiating hematomas from sarcomas. Beyond conventional T1- and T2-weighted sequences, diffusion-weighted imaging (DWI) and dynamic contrast-enhanced (DCE) MRI offer information on vascularity, necrosis, and cellular density [20]. Radiomics approaches, which extract quantitative imaging features beyond visual assessment, are being tested for their ability to predict malignancy with high accuracy [21]. Importantly, European Society of Musculoskeletal Radiology (ESSR) guidelines (2025) recommend that MRI should not be delayed when hematomas fail to resolve within 2–3 weeks or when red flags are present (mass > 5 cm, deep location, irregular margins). Yet adherence to these recommendations remains inconsistent, underscoring the need for decision-support tools [22].

Another frequent pitfall is delayed follow-up. The assumption that hematomas will resolve spontaneously within weeks may foster complacency, particularly in hemodynamically stable patients. Persistence of a mass beyond 2–3 weeks, especially when associated with ongoing symptoms or unchanged size, should prompt further investigation. Failure to do so risks delayed malignancy diagnosis, as documented in several case reports [14,23,24,25].

Resuming anticoagulation without thorough reassessment of a residual mass is problematic. In the presence of an undiagnosed tumor, renewed anticoagulation may trigger recurrent bleeding or intratumoral hemorrhage, complicating both diagnosis and treatment [26]. For these reasons, a more cautious and structured approach is warranted in the management of intramuscular hematomas in anticoagulated patients, one that integrates serial imaging, clinical reassessment, and timely biopsy when indicated.

Spontaneous muscular hematomas are relatively common, particularly in patients on anticoagulation or other antithrombotic therapies. However, the proportion of these hematomas that may actually represent an underlying tumor remains uncertain. Epidemiological data on this topic are limited, although recent real-world studies, such as the multicenter cohort by Bougeta et al. [4], provide detailed insights into the incidence, risk factors, and outcomes of major muscular hematomas in patients receiving antithrombotic agents. When considering tumors, it is important to distinguish between localized effects of a neoplasm causing bleeding and the systemic impact of cancer on hemostasis, such as hypercoagulability or coagulopathy. This broader perspective can help clinicians appropriately evaluate and monitor patients, balancing the likelihood of benign versus malignant etiologies. This case illustrates how an apparently uncomplicated hematoma in an anticoagulated elderly patient can mask a malignant neoplasm. It highlights the importance of early MRI and histologic evaluation in persistent or atypical lesions, even when anticoagulation parameters are therapeutic.

Based on the evidence and risk stratification criteria discussed above, we proposed a structured diagnostic algorithm for the evaluation and management of anticoagulated patients presenting with spontaneous muscular hematomas.

1. Initial Assessment
  - Evaluate patient history: anticoagulant type/dose, recent trauma, comorbidities.
  - Perform physical examination: size, location, pain, ecchymosis, functional impairment.
2. Risk Stratification
  - Low-risk features: small (<5 cm), superficial hematoma, recent minor trauma, resolving symptoms.
  - High-risk features/red flags: large (>5 cm), deep location, persistent or worsening pain, delayed resolution (>2–3 weeks), atypical MRI findings (solid enhancement, irregular margins, diffusion restriction).
3. Imaging Decision
  - Low-risk: clinical follow-up, ultrasound optional.
  - High-risk: MRI recommended to assess for atypical features; consider biopsy if suspicious for malignancy.
4. Follow-up and Monitoring
  - Monitor clinical progression every 1–2 weeks.
  - Reassess size, pain, and resolution of hematoma.
  - Escalate imaging or referral if the lesion fails to regress or red flags develop.
5. Oncologic Referral
  - Immediate referral for biopsy or specialist evaluation if red flags persist or imaging suggests malignancy.
6. Documentation and Risk Communication
  - Record anticoagulant therapy, clinical course, and follow-up plan.
  - Educate patient regarding signs that warrant urgent re-evaluation.

## 4. Literature Review: Malignancy Masquerading as Hematoma

This narrative review was conducted following the guidelines of Sukhera J. (2022) [5]. Literature searches were performed in PubMed, Scopus, and Web of Science using keywords: “spontaneous muscular hematoma,” “soft tissue tumor,” “differential diagnosis,” and “malignancy presenting as hematoma.” English-language articles from the last 15 years were included, focusing on clinical presentations, imaging features, and diagnostic strategies. Studies not relevant to human clinical cases or focusing solely on traumatic hematomas were excluded. Evidence was synthesized thematically to highlight differential diagnosis and clinical red flags.

While SMHs are generally considered benign complications of anticoagulation, their presentation can occasionally mimic soft tissue malignancies, especially when the evolution deviates from the expected course. A detailed comparison of typical clinical, radiological, and temporal characteristics can aid in distinguishing benign hematomas from malignant masses (Table 1). This differential process is crucial in elderly patients or those without clear traumatic triggers. Benign hematomas typically present suddenly following trauma or anticoagulant initiation, often with pain and ecchymosis, and resolve within 2–6 weeks. In contrast, malignant soft tissue tumors, such as undiagnosed sarcomas, may mimic hematomas but tend to grow progressively, persist for weeks, lack traumatic etiology, and exhibit atypical imaging features, including solid enhancement or irregular margins. Unrecognized warning signs may result in postponement of cancer diagnosis and appropriate management.

Moreover, several clinical and radiological red flags should prompt further diagnostic evaluation. These include a mass larger than 5 cm, deep anatomical location, persistent or worsening pain, and lack of hematoma resolution within 2–3 weeks [27,28,29]. In addition, atypical MRI features, such as solid enhancement, diffusion restriction, or irregular margins, may suggest a malignant process requiring biopsy (Table 2). Table 2 presents a proposed stratification system for evaluating SMHs that deviate from the expected clinical course. Incorporating such stratification into practice may support timely oncologic referral and reduce mismanagement related to attribution bias.

Additionally, neoplastic tissues, especially those in the gastrointestinal, genitourinary, and musculoskeletal systems, often harbor fragile vasculature prone to bleeding under anticoagulation, even within therapeutic ranges. In such cases, bleeding may not be a drug-related adverse event, but rather the first clinical manifestation of an undiagnosed malignancy. This “unmasking” phenomenon has been described in the literature and carries significant clinical implications [6].

### Gaps in Literature

Despite the increasing recognition of SMH in anticoagulated patients several important gaps remain, particularly when underlying malignancy is involved. No clinical guidelines currently provide specific recommendations for evaluating SMH in anticoagulated patients with red flags suggestive of malignancy, such as a persistent mass, disproportionate pain to the hematoma size, or lack of spontaneous resolution. This lack of guidance contributes to diagnostic delays, as case reports have shown bleeding being prematurely attributed to anticoagulation alone [7,8]. Validated imaging criteria for early identification of suspicious lesions are also lacking. Although MRI is the most accurate modality for soft tissue characterization, especially in differentiating benign hematomas from soft tissue sarcomas [9,10,11], no standardized early-phase MRI markers have been established. This often leads to inconsistent use of advanced imaging and missed opportunities for early diagnosis [12]. A further limitation lies in the absence of prospective comparative studies evaluating outcomes in patients with SMH who undergo early versus delayed biopsy. Without such data, clinical practice remains heterogeneous, and treatment decisions are based primarily on individual judgment rather than evidence. Similarly, the prognostic impact of anticoagulation resumption in patients diagnosed with sarcoma after SMH is poorly defined, creating uncertainty in balancing thrombosis and bleeding risks.

Additional gaps include the absence of prospective comparative studies evaluating early versus delayed biopsy, unclear prognostic implications of anticoagulation resumption after malignancy diagnosis, and lack of health economic evaluations regarding systematic imaging and biopsy. Currently, no validated diagnostic algorithms or risk scores exist to guide biopsy decisions, leaving clinical practice largely empirical [11,30]. Long-term outcomes of SMHs revealing malignancy, including recurrence, metastasis, and survival, remain poorly defined, as do anticoagulation management strategies in patients with concomitant soft tissue sarcoma [8,18,19,20]. Finally, there is no dedicated registry or multicenter observational cohort collecting prospective data on SMHs in anticoagulated patients. Establishing such a registry could substantially improve understanding of incidence, risk factors, diagnostic delays, and outcomes, while also providing a foundation for interventional studies and future clinical guidelines.

## 5. Future Directions

Given these gaps, future efforts should focus on systematic data collection, risk stratification, and development of diagnostic algorithms [6]. In our experience, patients who present with spontaneous, disproportionate, or anatomically unusual hemorrhage should undergo prompt and comprehensive evaluation, including imaging and, when appropriate, tissue diagnosis, to exclude underlying malignancy. Differentiating a spontaneous intramuscular hematoma from a soft tissue sarcoma can be clinically challenging, particularly in patients receiving oral anticoagulation therapy, where bleeding is often the presumed cause of any new mass or swelling. However, several clinical and radiological features can help distinguish a benign hematoma from an underlying malignancy. Prospective registries and multicenter collaborations are essential to define epidemiology, natural history and outcomes of SMHs, particularly those associated with malignancy [16,17]. Diagnostic algorithms that integrate clinical and radiologic red flags should be developed and validated to guide clinicians in real-time decision-making, ideally incorporating factors such as mass size, duration, depth, and INR levels [3]. Advanced imaging biomarkers, especially MRI-based parameters, need to be identified and validated to improve early differentiation between benign and malignant lesions [10]. Beyond imaging, translational research into circulating biomarkers, including cfDNA, microRNA signatures, and tumor-derived extracellular vesicles, holds promise for the non-invasive identification of malignancies presenting as hematomas [18]. Integration of these molecular tools with imaging could enable a precision-medicine approach to diagnosis. Furthermore, comparative outcome studies are warranted to assess the prognostic impact of underlying malignancy in MSH cases, including survival and recurrence rates [19]. Additionally, safe anticoagulation strategies must be investigated for patients in whom a tumor is identified after an MSH, as clinical decisions must balance the risk of recurrent bleeding with thrombotic complications [20]. Finally, health policy and cost-effectiveness research will be crucial to determine whether early MRI and biopsy protocols are justified compared to the costs and morbidity of delayed cancer diagnosis. Such analyses could provide the economic rationale needed to implement systematic diagnostic strategies on a wider scale [21,22,23].

## 6. Conclusions

In patients receiving anticoagulant therapy, new soft tissue masses should not be automatically attributed to bleeding complications. Malignancy must be considered, particularly when symptoms persist or progress. A thorough diagnostic workup, including imaging and biopsy, is essential to avoid delays in receiving appropriate treatment. This case reinforces the importance of a high index of suspicion for neoplastic processes in anticoagulated patients with unusual or unresponsive musculoskeletal symptoms. Clinicians should therefore adopt a systematic approach that moves beyond attribution bias. The integration of early MRI, careful follow-up and timely biopsy of non-resolving lesions could markedly reduce diagnostic delays. By viewing hematomas not only as benign consequences of anticoagulation but also as potential “red flag” events, physicians can better balance the competing risks of bleeding and missed malignancy. Looking forward, the establishment of multicenter registries, the validation of imaging biomarkers, and the exploration of circulating molecular markers will be critical to refine diagnostic accuracy and to personalize management strategies. Until such tools are available, clinical vigilance remains the cornerstone: every atypical hematoma should be considered not just a complication but an opportunity for earlier cancer detection and intervention.

## Figures and Tables

**Table 1:** Key Differences Between Benign Spontaneous Intramuscular Hematomas and Malignant Soft Tissue Masses Mimicking Hematomas.

Feature	Benign Hematoma	Malignant Tumor (e.g., Soft Tissue Sarcoma)
Onset	Sudden, often post-traumatic or anticoagulation-related	Gradually, sometimes with vague discomfort
Growth pattern	Rapid initial swelling with stabilization or resolution	Progressive, enlarging over weeks/months
Pain	Usually acute and resolves	May be dull, persistent, or increase over time
Skin changes	Ecchymosis, or bruising, is typical	May be absent or delayed
Response to therapy	Tends to resolve with conservative management	Persist or worsen despite treatment
Laboratory findings	Usually normal; minor elevation in inflammatory markers possible	May show elevated CRP, LDH or tumor markers (dependig on histotype)
Imaging: Ultrasound	Hypoechoic/heterogeneous with fluid levels	Solid mass, possible internal vascularity
Imaging: MRI	Homogeneous hematoma; evolution over time	Heterogeneous, irregular margins, enhancement
Follow-up evolution	Shrinks or disappears in 2–6 weeks	Remains stable or grows
Need for biopsy	Rare if clinical/imaging supports hematoma	Indicated if mass persists > 3–4 weeks or atypical
Common oversight	Attribution to anticoagulation	Mistaken for hematoma, delaying diagnosis
Tumor histotypes	N/A	Malignant fibrous histiocytoma/myxofibrosarcoma ~40%, followed by pleomorphic sarcoma and Ewing sarcoma

Legend: Hematomas are typically self-limiting. Persistent, enlarging, or atypical lesions warrant re-evaluation and possible biopsy.

**Table 2:** Proposed Diagnostic Red Flags and Stratification in SMHs Suggesting Possible Underlying Malignancy.

Clinical or Imaging Feature	Diagnostic Implication	Suggested Action
Mass > 5 cm	Potential for soft tissue sarcoma	MRI with contrast
Deep location (e.g., intramuscular, retroperitoneal)	Less accessible for clinical assessment	MRI preferred over US
Lack of resolution after 2–3 weeks	Suspicion of neoplasm	Repeat imaging, consider biopsy
Absence of trauma or INR < therapeutic range	Hematoma is less likely to be anticoagulation-related	Search for alternative causes
Pain persistence or progression	Suggests mass effect or infiltration	Further imaging and clinical evaluation
Atypical MRI findings (e.g., enhancement, irregular borders)	May indicate malignancy	Radiologic review and biopsy
Previous history of cancer or sarcoma	Risk of recurrence/metastasis	Oncology consult; tissue diagnosis
Recurrence at the same site	Sarcoma or local invasion	Biopsy and staging work-up

Legend: Any combination of these red flags should prompt reassessment and referral for oncologic evaluation.

## Data Availability

No new data were created or analyzed in this study.

## Abbreviations

The following abbreviations are used in this manuscript:

SMHs.	Spontaneous intramuscular hematomas.
OAT	Oral anticoagulation therapy
VKA	Vitamin K antagonist
